# The effects of camel chymosin and *Withania coagulans* extract on camel and bovine milk cheeses

**DOI:** 10.1038/s41598-021-92797-6

**Published:** 2021-06-30

**Authors:** Mustapha Mbye, Huda Mohamed, Abdul Raziq, Afaf Kamal-Eldin

**Affiliations:** 1 Department of Food Science, College of Food and Agriculture, United Arab Emirates University, Al Ain, PO Box 15551, Abu Dhabi, United Arab Emirates; 2 Al-Ain Farms for Livestock Production, Al Ain Dubai Road, Al Ain, United Arab Emirates; 3 Department of Food, Nutrition and Health, College of Food and Agriculture, United Arab Emirates University, Al-Ain, United Arab Emirates

**Keywords:** Biochemistry, Biotechnology

## Abstract

*Withania coagulans* (*W. coagulans*) extract and camel chymosin have aspartic protease capable of coagulating milk for cheese production. This study investigated the quality of camel and bovine milk cheeses coagulated using *Withania* extracts, came chymosin, and their mixture in two experiments. In Experiment (1), a factorial design with four factors (*W. coagulans*, camel chymosin, incubation time, and incubation temperature) was performed. The effect of these factors on cheese’s yield and hardness were assessed. An enzyme concentration corresponding to a 36 µg/L of milk of *W. coagulans*, 50 IMCU/L of camel chymosin, holding time of 4 h, and incubation temperature of 60 °C provided the optimal textural hardness for both camel and bovine milk cheeses. Seven treatments were analyzed in experiment (2) were analyzed for physicochemical properties, yield, and sodium dodecyl sulfate–polyacrylamide gel electrophoresis (SDS-PAGEitation). The results showed that pure Withania extract exhibited the lower coagulating effect resulting in cheeses with low yield, hardness, fat, protein, and total solids. The SDS-PAGE electropherograms of camel cheese showed several low molecular weight bands as compared to bovine cheese. This phenomenon is due to excessive proteolysis in camel cheese, which we believed is caused by the presence of endogenous enzymes.

## Introduction

The production and consumption of camel milk (CM) have increased over the years. The global production of camel milk and its products is projected to increase to 7% between 2018 and 2027^1,2^. CM is acknowledged for its nutritional and therapeutic qualities^3^. Despite the exceptionality, of CM, it's technically more difficult to process into cheese^4–7^. The difficulty in producing high-quality cheeses from CM is attributed to larger casein micelles size^8,9^, long coagulation time^10^, low amount of κ casein^11–13^, the small size of fat globules^14^ compared to bovine milk.

Production of cheese from camel milk has been challenging due to the lack of coagulants that can specifically cleave camel milk κ casein. Thus, numerous approaches to make cheese from camel milk have been studied, some of these studies include the use of camel chymosin with started cultures for acidification and better curd quality^15–22^. In addition to the use of chymosin, there has been a steady growth in the use of plant enzymes in the cheese industry because they are readily accessible and have simple extraction and refining processes^23^. Furthermore, animal enzymes are becoming unpopular in some countries due to diet and religious matters^24^. Plant proteases can be obtained from extracts of *Cynara cardunculus*^25^, Algerian spontaneous *Cynara cardunculus*^26^, artichoke (*Cynara scolymus*, L.)^27^, crude extracts of ginger rhizome (*Zingiber officinale*)^28^, and *Withania coagulans *(*W. coagulans*)^29–31^. *W. coagulans* belongs to the family Solanaceae and grows in Afghanistan, Pakistan, India, and Iran. Its extract has been traditionally used as a substitute for rennet in the preparation of cottage cheeses from bovine, goat, and sheep milk especially in Baluchistan, Pakistan^32,33^. The active proteolytic enzyme in Withania coagulans was estimated to have a molecular weight of 66 KDa optimum activity at 70 °C and pH 4^30,34^. The high proteolytic nature of most plant proteases may result in bitter flavors and low cheese yields; thus, their use is limited in cheese production^35,36^.

It is believed a mixture of plant-based enzymes and camel chymosin may enhance milk coagulation and limit some negative effects caused by the use of plant enzymes alone and lead to an improvement of the quality and yield of cheese. Thus, this work aimed to conduct a comparative study of camel and bovine milk cheese coagulated with *W. coagulans* extract, camel chymosin, and mixtures of the two enzymes to explore the differences between the two milk sources. Two experiments were performed. In the first experiment, the effect of four factors i.e. (*Withania coagulans* concentration, camel chymosin concentration, incubation time, and incubation temperature) cheese’s yield and hardness was assessed. In the second experiment, the effect of the two individual enzymes and five mixtures thereof on yield, physicochemical parameters, and sodium dodecyl sulfate–polyacrylamide gel electrophoresis (SDS-PAGE) profiles of the proteins of cheese and whey were analyzed.

## Materials and methods

### Materials

The milk used in cheese preparation pooled raw camel milk from 300 camels and bovine milk from 800 bovines and was obtained from Al Ain Dairy farm, Al Ain City, Emirates of Abu Dhabi, UAE. The milk samples were delivered to the Food Science Department at United Arab Emirates University in refrigerated coolers (4 °C). Milk composition was as follows: camel milk (pH, 6.5; acidity, 0.16; total solids, 12.4%; protein, 2.8%, and fat, 3.3%) and bovine milk (pH, 6.69; acidity, 0.16%; total solids, 12.5%; protein, 3%, and fat, 3.4%).

The lyophilized yogurt starter culture Yoflex Express^®^ 1.0 (1:1) mixture of *Streptococcus thermophilus* and *Lactobacillus bulgaricus* subsp. *delbrückii* was used. Recombinant camel chymosin (CHY-MAX^®^M), with an activity of 1000 IMCU/mL, was kindly provided by Chr. Hansen Denmark. Fresh extracts from *W. coagulans* seeds, obtained from Loralai, Balochistan, Pakistan, were used. Urea Bio Ultra (for molecular biology, > 99%), N, N, N′, N′-Tetramethylethylenediamine (TEMED), calcium chloride, and all other chemicals and reagents, all of the analytical grade, were purchased from Sigma-Aldrich (St. Louis, Missouri, USA). Precision Plus Protein–unstained standard (molecular weight marker), 4 × Laemmli sample buffer (62.5 mM Tris HCl, pH 6.8, 10% glycerol, 1% lithium dodecyl sulfate, 0.005% bromophenol blue), resolving gel buffer (1.5 M Tris HCl, pH 8.8), stacking gel buffer (0.5 M Tris HCl, pH 6), SDS solution (10%), dithiothreitol (DTT), ammonium persulphate (APS), 10 × TGS buffer (0.25 M Tris, 1.92 M glycine, and 1% sodium dodecyl sulfate), QC Colloidal Coomassie stain, and 30% acrylamide/Bis solution (29:1, v/v) were purchased from Bio-Rad Laboratories Inc. (Hercules, California, USA).

### Experimental design

The first experiment was performed using a central composite rotatable design, with varying combinations of *W. coagulans* concentration (7, 21, 36, 50, and 65 µg/1000 mL milk), camel chymosin concentration (10, 30, 50, 70, and 90 IMCU/1000 mL milk), incubation time, (1, 2, 4, 6 and 8 h), and incubation temperature (40, 50, 60, 70 and 80 °C), which were independent variables; then, the response variables (cheese yield and hardness) were measured (Table 1). In the second experiment, which was performed in triplicate, three sets of cheeses were made from each treatment using pure *Withania* extract, pure camel chymosin, or their mixture, as shown in Table 2. In this experiment, several other traits were measured in addition to yield and hardness, including cheese color, titratable acidity/pH, protein, fat content, and SDS-PAGE electrophoretic profiles of both camel and bovine cheese, whey, and milk.

**Table 1:** Experimental design of the independent variables (enzyme concentration, incubation time, and temperature) and results of the associated response variables (cheese yield and hardness).

Run order	Independent variables				Response variables			
	*W. coagulans* extract (µg protein/1000 mL milk)	Chymosin (IMCU/1000 mL milk)	Incubation time (h)	Incubation temperature (°C)	Yield (%)		Hardness (g)	
					Camel cheese	Bovine cheese	Camel cheese	Bovine cheese
1	65	50	4	60	14.66	11.88	445	1302
2	36	50	4	60	12.98	8.44	609	1769
3	36	50	4	40	14.87	10.23	431	1396
4	50	70	6	70	16.76	14.02	260	799
5	21	30	6	70	15.08	12.75	398	1245
6	21	30	6	50	15.23	12.59	392	1168
7	7	50	4	60	14.51	11.01	491	1424
8	50	30	2	70	15.55	12.88	378	1009
9	21	70	6	70	16.71	13.89	258	844
10	21	30	2	50	13.13	8.78	580	1752
11	36	50	4	60	14.36	10.95	498	1490
12	36	50	4	60	13.44	9.54	561	1645
13	36	10	4	60	16.47	14.23	259	781
14	50	30	2	50	16.33	13.98	261	860
15	21	70	2	70	16.02	13.72	299	909
16	36	50	0	60	16.78	14.45	254	770
17	21	70	2	50	15.52	12.91	382	1009
18	36	50	4	60	13.89	9.88	511	1567
19	36	50	4	60	14.02	10.8	501	1555
20	21	30	2	70	15.4	12.87	391	1156
21	36	50	4	80	15.06	12.98	354	988
22	50	30	6	50	15.03	12.56	400	1250
23	36	50	4	60	12.89	7.03	671	2297
24	50	30	6	70	15.71	13.01	345	940
25	36	50	8	60	14.96	11.44	411	1344
26	36	50	4	60	14.91	11.61	426	1336
27	36	90	4	60	16.66	13.87	276	876
28	21	70	6	50	15.92	13.62	309	918
29	50	70	2	50	15.87	13.57	311	925
30	50	70	2	70	15.81	13.34	322	931
31	50	70	6	50	16.22	13.88	287	895

**Table 2:** *Withania* and chymosin enzyme mixing protocols for detailed studies on cheese characteristics*.

Treatment	Abbreviation	*Withania *(µg/1000 mL of milk)	Chymosin (IMCU/1000 mL of milk)
Pure *Withania*	PW	65	0
Pure Chymosin	PC	0	70
Low *Withania*–Low Chymosin	LWLC	7	10
Low *Withania*–High Chymosin	LWHC	7	70
High *Withania*–Low Chymosin	HWLC	65	10
Medium *Withania*–Medium Chymosin	MWMC	36	40
High *Withania*–High Chymosin	HWHC	65	70

*Experiments were performed at a fixed temperature (60 °C) and incubation time (4 h).

### Enzyme extraction

Enzymes were extracted from *W. coagulans* following a previously described method^34^. *W. coagulans* berries were washed and dried in a cool place and then ground. The powder (10 g) was mixed with 100 mL of 1% saline solution for 24 h at 4 °C with agitation. The mixture was centrifuged at 9000×*g* at 4 °C for 30 min. The supernatant was filtered through Whatman paper No. 1^30^. The protein content in *W coagulans* crude extract was determined following Bradford method^37^ using bovine serum albumin (BSA) as a standard. The absorbance of the supernatant was measured at 595 using a UV/visible spectrophotometer (Pharmacia Biothch ultrospect 3000, Cambridge, England). A freshly crude extract was used in making the cheeses.

### Cheese preparation

One litter of raw camel or bovine milk was processed into cheeses, in each of three repetitions per treatment, using camel chymosin or *W. coagulans*, or the mixture of the two coagulans. The milk was heated to 63 °C for 30 min and calcium chloride (3%) was added based on earlier reports^12^. The milk's temperature was brought down to 43 °C and it was inoculated with 3% (w⁄v) of an active thermophilic yogurt starter culture Yoflex Express^®^ 1.0 (*Streptococcus thermophilus* and *Lactobacillus delbrueckii* subsp. *bulgaricus*). The incubation continued for 60 min until the milk's pH was lowered to 6.2, and then the camel chymosin (CHY-MAX^®^M), *W. coagulans* extract, or the mixture was added to the milk and stirred thoroughly^38^. The enzyme concentrations, incubation time, and incubation temperature in Experiment (1) are given in Table 1. While in Experiment (2) after the addition of (3%) starter culture and 50 IMCU/L of milk camel chymosin or 36 µg/L of milk or the mixture. The milk was incubated at room temperature (25 °C) for four hours until the pH reached 4.8, and the firm curd was observed, and then the curd was placed in cheesecloth to drain for 8 h^16,22,39^.

### Cheese yield and physicochemical properties

Fresh cheese prepared from 1 L of milk per trial was weighed, after 8 h of draining, using Metis digital weighing scale (Dubai, UAE). The cheese yield was calculated as the percentage of the weight of the fresh cheese as follows (Yield = kg of fresh cheese × 100/mL of processed milk)^40^. The pH of the samples from both experiments was determined using a digital pH meter (Starter3100; Ohaus, New Jersey, USA), and the titratable acidity was determined in triplicate using the standard method ISO/TS 11869:2012 (IDF/RM 150:2012)^22^. The seven treatments of camel and bovine cheeses and wheys in experiment (2) were evaluated for their contents of fat, protein, and total solid using a Near-Infrared Multipurpose Analyzer (Bruker Optik GmbH, Ettlingen, Germany). All the samples were tested on the same day, with each sample analyzed in triplicate. The texture profile analysis (TPA) of the camel and bovine cheeses from both experiments was analyzed for textural hardness using a CT III texture analyzer equipped with a 4.5-kg load cell (Brook-field, Middleboro, Massachusetts, USA). TPA was performed with a compression test of the cheese in a 40-mL cup using a 25-mm-diameter perplex cylindrical probe (TA11/1000) with a test speed of 2 mm/s and target distance of 5 mm^22^. The hardness pattern (force–time) was analyzed in triplicate. The color characteristics of camel and bovine cheeses in experiment (2) were measured using a HunterLab color analyzer (Mini Scan XE Plus, Model 45/0-S, Hunter Lab Inc., Reston, Virginia, USA). Color values, L*, a*, and b*, were recorded, with each value being the average of four measurements. This color system comprises a lightness component (L*), a* component for green (− a) to red (+ a), and a b* component from blue (− b) to yellow (+ b). Fourier-transform infrared (FTIR) spectroscopy of the cheese samples from experiment (2) was performed using an ATR-FTIR Spectrometer (Nicole™ 1S50 FTIR; Thermo Fisher Scientific, Massachusetts, USA) at mid-infrared wavelengths. The infrared spectrum was recorded between 400 and 4000 cm^−1^ at a resolution of 4 cm^−1^. To improve the signal-to-noise ratio, 124 scans were used per spectrum. Three spectra were taken from each sample.

### SDS-PAGE of cheeses and whey proteins

Proteolytic activity of the seven treatments of camel and bovine cheese, whey, and milk samples from camel and bovine in Experiment (2) were analyzed using SDS-PAGE. Cheese samples were prepared using previously reported methods^21,41^. The fresh cheese samples (0.6 g) were dissolved in 25 mL of 8 M urea and whey samples (0.6 g) were dissolved in 8.3 mL of 8 M urea. The samples were homogenized for 2 min using T 25 digital Ultra-Turrax (IKA-Werke GmbH and Co. KG, Staufen, Germany). For the complete solubilization of caseins, the sample and urea mixtures were incubated in a temperature-controlled water bath at 37 °C for 2 h. The cheese and urea mixture was defatted by centrifugation at 9150×*g* at 4 °C for 35 min. The solution was filtered through Whatman no. 1 filter paper (pore size, 11 µm). 10 µL of the filtered sample was added to 30 µL of 4 × Lamelli buffer solution containing 50 mM Dithiothreitol (added freshly). The sample and sample buffer mixture were heated in a temperature-controlled water bath for 5 min at 90 °C. From this mix, 6 µL was loaded on the hand-casted polyacrylamide gels. Electrophoresis was performed at 200 V using a power supply from PowerPac™ Basic Power supply (Bio-Rad Laboratories Inc., Hercules, California, USA). The running buffer (pH = 8.3) used was a 10 × Tris/Glycine/Sodium dodecyl sulphate Buffer (25 mM Tris, 192 mM glycine, and 0.1% (w/v) sodium dodecyl sulphate.

Gels with 1 mm thickness were prepared using the gel hand casting accessories provided with the Bio-Rad Mini-PROTEAN Tetra cell (Bio-Rad Laboratories Inc., Hercules, California, USA). A 12% resolving gel and 4% stacking gel were prepared. To prepare a quantity of 15 mL of 12% resolving gel solution the following were added: 6 mL 30% acrylamide/Bis Solution 29:1, 3.75 mL 1.5 M Tris HCl (pH 8.8), 150 μL of SDS solution 10% (w/v), 5.03 mL deionized water, 75 μL of 10% APS (ammonium persulphate), 7.5 μL N, N, N′, N′-Tetramethylethylenediamine. To prepare a quantity of 15 mL of 4% stacking gel solution the following were added: 1.98 mL 30% acrylamide/Bis Solution (29:1, v/v), 3.78 mL 0.5 M Tris HCl (pH 6.8), 150 μL SDS solution 10% (w/v), 9 mL deionized water, 75 μL 10% APS, 15 μL TEMED. The gels were kept for one hour in a solution of 40% ethanol and 10% acetic acid for fixation of the protein bands. Gels were stained for 20 h using the QC colloidal Coomassie stain. The gels were de-stained for three hours by changing the distilled water three times. Gels image acquisition and densitometry were performed by Gel Doc™ XR+ and Chemidoc™ XRS+ Imaging Systems (Bio-Rad Laboratories Inc., Hercules, California, USA).

For imaging the gels, a UV/White light conversion screen was used. The instrument was operated by the Image lab software (Bio-Rad Laboratories Inc., Hercules, California, USA). The software was used to determine the protein bands' molecular weights, integrate the peaks, and determine their relative densities.

### Statistical analysis

A full factorial central composite design was used in experiment (1). The values of four independent factors (*W. coagulans* extract concentration, camel chymosin concentration, incubation time, and incubation temperature) and their response variable are shown in (Table 1). The design consisted of 31 experiments including 7 central point repetitions that would account for the error in the model. This experiment was designed using Minitab^®^19 (Connecticut, USA). The model design was fitted to each response using the following equation:

$$ y = \beta _{0}  + \sum\limits_{{i = 1}}^{4} {\beta _{i} } x_{i}  + \sum\limits_{{i = 1}}^{4} {\beta _{{ii}} } x_{i}^{2}  + \sum\limits_{{i = 1}}^{3} {\sum\limits_{{j = i + 1}}^{4} {\beta _{{ij}} } } x_{i} x_{j} $$

where y is the response, β_0_ is a constant, β_i_ is the linear coefficient, β_ii_ is the quadratic coefficient, and β_ij_ is the interaction coefficient. X_i_ and X_j_ are two independent variables. In this experiment, raw data from freshly extracted cheese was analysed.

In the second experiment, the physicochemical, and yield from freshly extracted cheese were analyzed in triplicate, and the mean values were used in the calculations. The statistical analysis was performed using IBM SPSS (SPSS Inc., Chicago, Illinois, USA). Data were analyzed using a one-way analysis of variance. The results were presented as the mean values of triplicate and their standard deviations. Mean values were compared using the least significant difference test, and p ≤ 0.05 was considered to represent statistical significance.

## Results and discussion

### Effects of camel chymosin and *Withania coagulans* on the yield and hardness of camel and bovine cheeses

**Figure 1:**
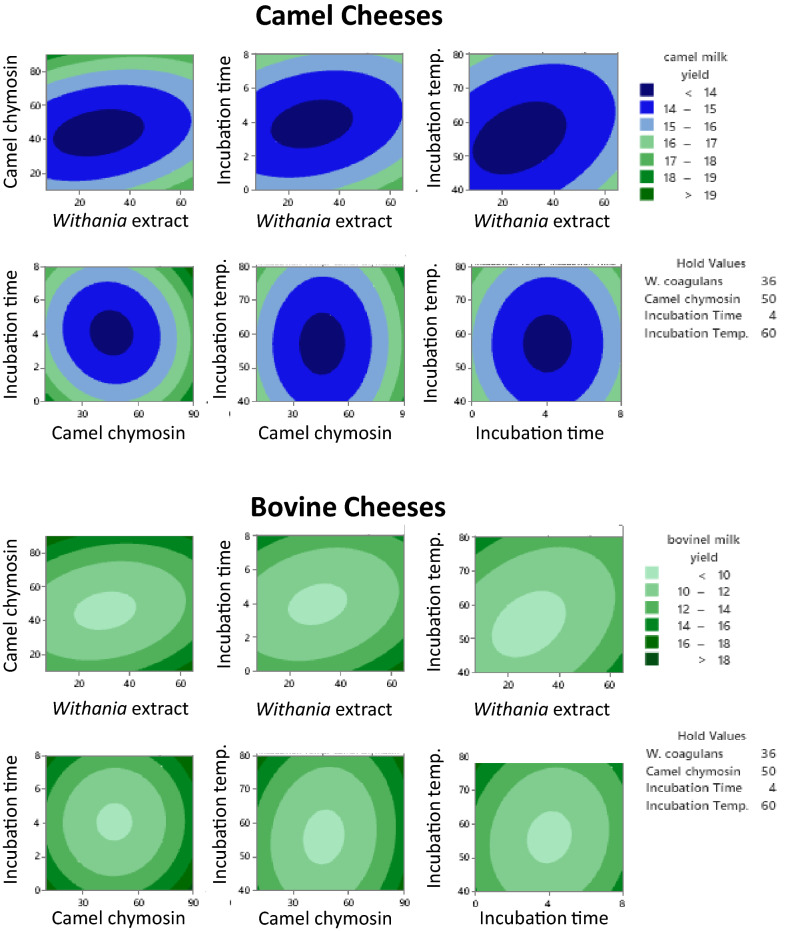
Interaction effects of four independent variables on the yield of camel and bovine milk cheeses: camel chymosin (IMCU/1000 mL milk), *Withania* extract (µg protein/1000 mL milk), Incubation time (hours), and Incubation temperature (°C) (Experiment 1).

**Figure 2:**
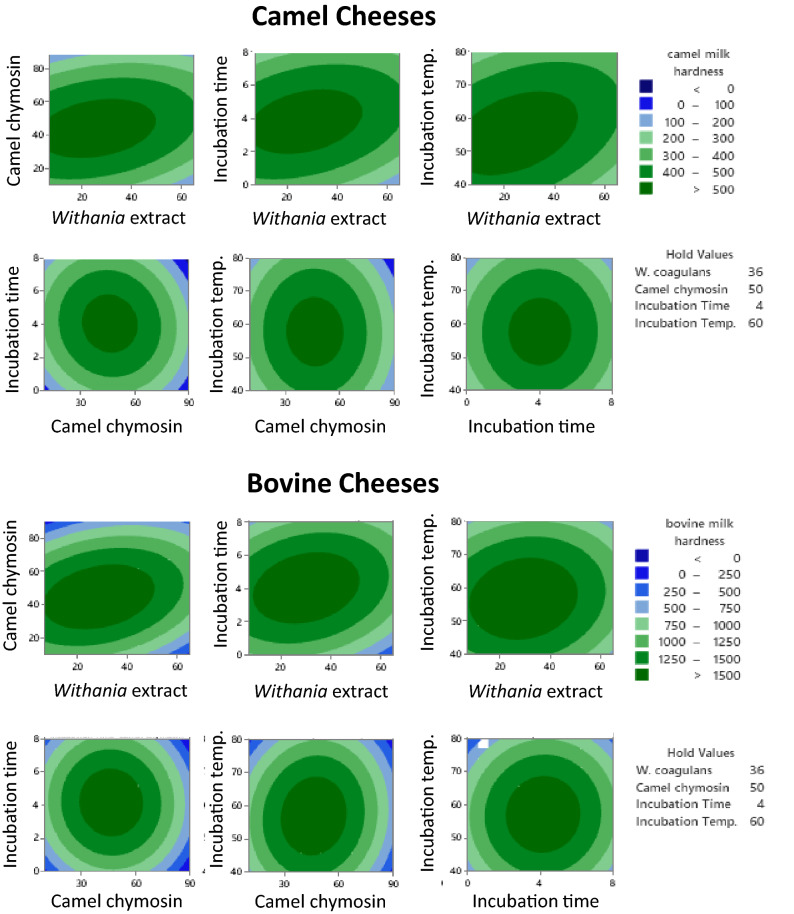
Interaction effects of four independent variables on the hardness of camel and bovine milk cheeses: camel chymosin (IMCU/1000 mL milk), *Withania* extract (µg protein/1000 mL milk), Incubation time (hours), and Incubation temperature (°C) (Experiment 1).

**Figure 3:**
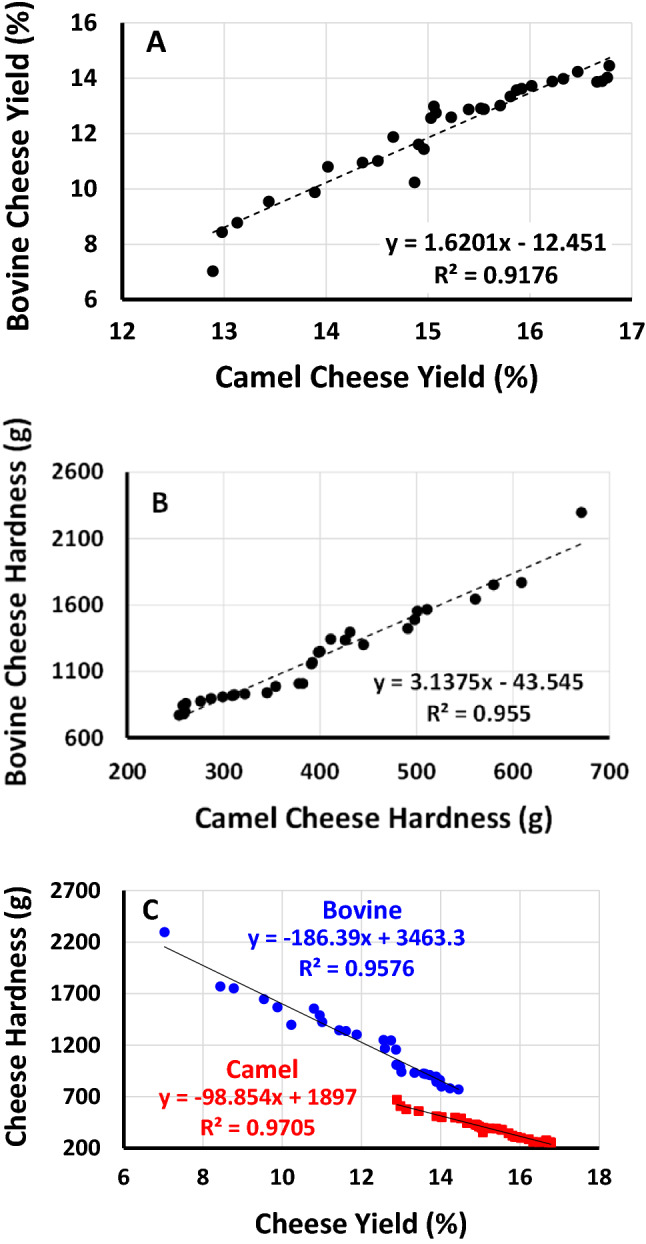
Correlations between (**a**) camel and bovine cheese yield (%), (**b**) camel and bovine cheese hardness (g), and (**c**) cheese yield and cheese hardness for camel (red) and bovine (blue) cheeses. All correlations are significant (p < 0.001). The experimental design is presented in Table 1 (Experiment 1).

Table 1 presents the experimental design for the independent variables (*W. coagulans* concentration, camel chymosin concentration, incubation time, and incubation temperature) and the results of two associated response variables (cheese yield and hardness) in experiment (1). Among the different combinations, crude extracts of *W. coagulans* (36 µg/L milk), camel chymosin (50 IMUC/L milk), coagulation time (4 h), and incubation temperature (60 °C) were the optimum conditions providing the lower fresh yield (Fig. 1) and the highest hardness (Fig. 2) for both camel and bovine milk cheeses. The negative correlation between the fresh yield and the hardness of both camel and bovine cheeses (p < 0.001) as shown in Fig. 3 is consistent with our previous observations and is explained by the increased retention of moisture in soft cheeses^22^. As the concentrations of added enzyme and the incubation temperature and time exceeded the optimum concentrations, the cheese started to become softer in agreement with literature^27^. This decrease in hardness may be explained by increased proteolysis of the casein proteins^22,43,44^.

Table 3 presents mathematical models that indicate the significance of the independent variables and their interactions in affecting the yield and hardness of camel and bovine cheeses in experiment (1). All models significantly (p < 0.05) suggest that the independent variables (concentration of *W. coagulans* and camel chymosin, and incubation time and temperature) collectively have similar effects on the yield and hardness of both camel and bovine cheese although the magnitude of the effect is different. However, higher yield and lower hardness are noticed more in camel cheeses than bovine cheeses^42^. The results also show that chymosin interactions are more important than those of *Withania* in terms of affecting cheese yield and hardness. The significant terms in the models were the constants related to the milks and the quadratic terms C*C, TP*TP, and TM*TM, all contributing negatively to the hardness of camel and bovine cheeses. These models suggest that the independent variables affected the camel and bovine milk cheeses fresh yield and hardness in similar ways but the magnitude of the effects was different due to differences between the two milks.

**Table 3:** Model for the relationships between dependent and independent variables and estimated regression coefficients and their significance*.

Model constants and coefficients	Yield (%)		Hardness (g)	
	Camel milk cheese	Bovine milk cheese	Camel milk cheese	Bovine milk cheese
Constant	+ 26.39***	+ 25.80***	− 939***	− 3298***
C1 (W)	+ 5.28	+ 7.1	+ 713	+ 369
C2 (C)	− 0.014	− 0.018	+ 1.519	+ 3.55
C3 (TM)	− 1.016	− 0.90	+ 91.9	+ 264
C4 (TP)	− 0.314	− 0.449	+ 40.3	+ 134
C5 (W*W)	+ 5.62	+ 12.73	− 517	− 2191
C6 (C*C)	+ 0.006***	+ 0.000***	− 0.001***	− 0.005***
C7 (TP*TP)	+ 0.13**	+ 0.221**	− 13.64**	− 41***
C8 (TM*TM)	+ 0.003*	+ 0.005*	− 0.396**	− 1.304**
C9 (W*C)	− 0.005	− 0.007	+ 0.483	+ 1.77
C10 (W*TP)	− 0.423	− 0.761	+ 49.2	+ 127
C11 (W*TM)	− 0.094	− 0.190	+ 11.34	+ 13.8
C12 (C*TP)	+ 0.000	− 0.000	− 0.019	− 0.023
C13 (C*TM)	− 0.000	− 0.008	− 0.001	+ 0.013
C14 (TP*TM)	+ 0.002	− 0.008	+ 0.053	+ 0.43
Model p-value	0.01**	0.02*	0.009**	0.02*

*C, chymosin; W, *W *coagulant; TM, time; TP, temperature. Significance of model parameters: *p < 0.05, **p < 0.01, and ***p < 0.005.

**Figure 4:**
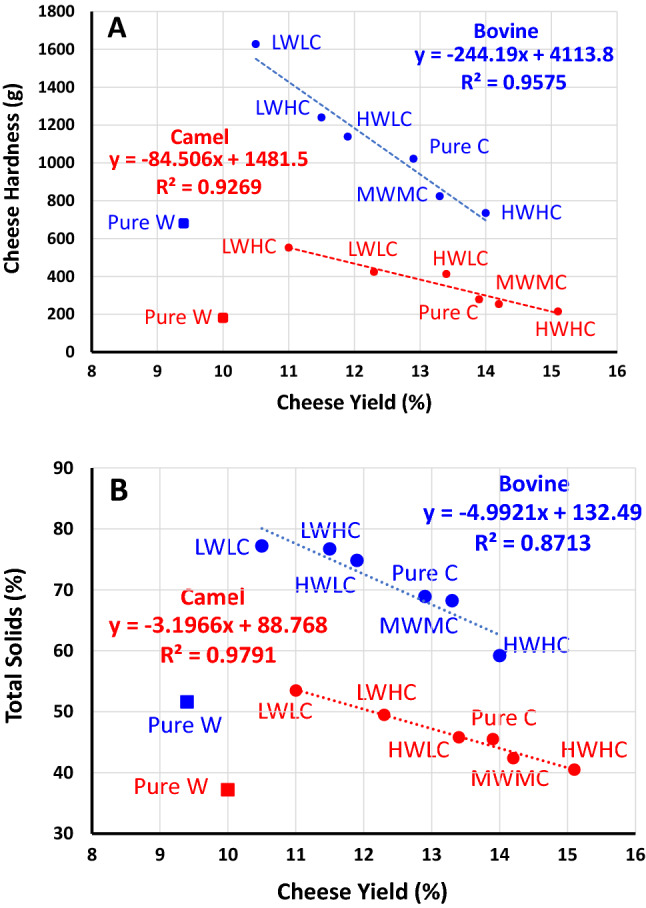
Correlations between (**a**) cheese yield and cheese hardness and between (**b**) cheese yield and cheese total solids for camel (red) and bovine (blue) cheeses. Correlations excluding pure *Withania* treatments are significant for both camel and bovine cheeses (p < 0.001). The codes are shown in Table 2 (Experiment 2).

The second experiment, based on seven treatments Table 2, was performed by combining different concentrations of chymosin and *W. coagulans* extracts at fixed incubation temperature (60 °C) and incubation time (4 h) that were chosen based on the results of Experiment 1. Table 4 presents results of the yield, hardness, and color of camel and bovine milk cheeses prepared using *W. coagulans* extracts, pure chymosin, and their mixtures. Cheese made from pure *W. coagulans* alone has the lowest cheese yield and hardness and is more yellow compared to the other cheeses. This yellow color of the *Withania*-treated cheeses may be the result of the presence of some water-soluble compounds in the berries^31^. The low yield and hardness of cheeses coagulated with the *W. coagulans* extract could be associated with poor coagulating properties of its protease compared with chymosin. The mixture of low camel chymosin and low Withania extract produced cheeses of higher hardness and total solids from both camel and bovine milks (Fig. 4). In agreement with the results of Experiment 1, the camel chymosin was more crucial for the cheese yield and hardness than the Withania extract. However, as the concentration of either enzyme increased, the cheeses became soft and fragile with high moisture content due to excessive hydrolysis of caseins^22,43,44,45,46^.

**Table 4:** Yield, hardness, and color of camel and bovine milk cheeses*

Treatment**	Yield (%)	Hardness (g)	Color		
			L*	a*	b*
**Camel milk cheese**					
PW	10.0 ± 0.75^f^	181 ± 6.66^e^	83.2 ± 0.2^e^	− 1.5 ± 0.09^b^	14.7 ± 0.31^a^
PC	13.9 ± 0.17^b^	279 ± 5.29^c^	92.3 ± 0.3^a^	− 1.7 ± 0.10^d^	6.7 ± 0.06^f^
LWLC	11.0 ± 0.16^e^	552 ± 19.5^a^	89.6 ± 0.3^c^	− 1.7 ± 0.01^e^	8.3 ± 0.19^e^
LWHC	12.3 ± 0.31^d^	424 ± 9.85^b^	91.3 ± 0.3^b^	− 0.9 ± 0.01^a^	5.8 ± 0.05^ g^
HWLC	13.4 ± 0.34^c^	413 ± 8.33^b^	83.6 ± 0.4^e^	− 1.6 ± 0.02^c^	13.9 ± 0.09^b^
MWMC	14.2 ± 0.22^b^	254 ± 8.14^c^	89.4 ± 0.1^c^	− 1.3 ± 0.03^b^	9.9 ± 0.05^c^
HWHC	15.1 ± 0.36^a^	215 ± 17.3^d^	88.6 ± 0.2^d^	− 1.7 ± 0.03^d^	9.1 ± 0.11^d^
**Bovine milk cheese**					
PW	9.4 ± 0.98^e^	681 ± 55^f^	79.9 ± 0.03^g^	0.5 ± 0.03^b^	22 ± 0.06^a^
PC	12.9 ± 0.09^b^	1022 ± 35^d^	90.7 ± 0.07^a^	− 1.2 ± 0.03^f^	8.9 ± 0.11^e^
LWLC	10.5 ± 0.2^d^	1628 ± 18^a^	88.7 ± 0.04^c^	0.8 ± 0.03^a^	11.7 ± 0.08^c^
LWHC	11.5 ± 0.47^c^	1240 ± 27^b^	89.7 ± 0.03^b^	0.8 ± 0.02^a^	9 ± 0.11^e^
HWLC	11.9 ± 0.24^c^	1139 ± 35^c^	85.9 ± 0.05^f^	− 0.1 ± 0.03^c^	12.3 ± 0.14^b^
MWMC	13.3 ± 0.3^b^	824 ± 24^e^	88.5 ± 0.04^d^	− 0.9 ± 0.03^e^	10.3 ± 0.23^d^
HWHC	14.0 ± 0.3^a^	735 ± 36^ef^	86.5 ± 0.03^e^	− 0.8 ± 0.03^d^	11.7 ± 0.13^c^

*Comparison was made between the different treatments for each cheese. Values within each column and cheese category (camel milk cheese or bovine milk cheese) carrying different superscripts are statistically different (p < 0.05, n = 3 per treatment).

**Abbreviations are shown in Table 2.

**Figure 5:**
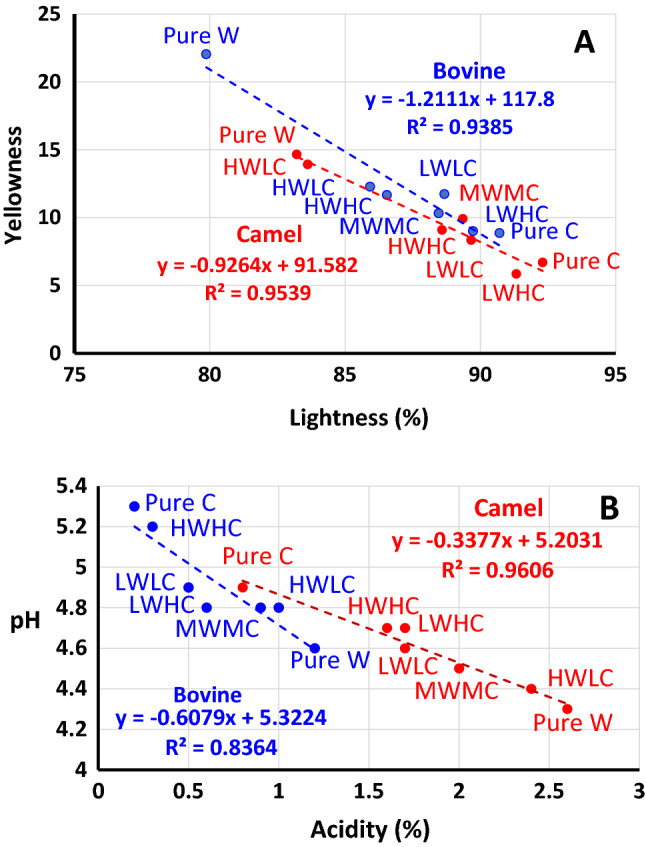
Correlations for camel (red) and bovine (blue) cheeses between (**a**) lightness and yellowness (CM cheeses are lighter in color and less yellow than BM cheeses; p < 0.001) and between (**b**) acidity and pH (CM cheeses have higher acidity and lower pH than BM cheeses; p < 0.001). All correlations are significant (p < 0.001). Chymosin yields cheeses that are less yellow, lighter in color, less acidic, and with higher pH than those yielded with *Withania* treatment (p < 0.001). The codes are presented in Table 2 (Experiment 2).

Thus, the results from the two experiments showed that unripened cheeses produced from camel milk have higher moisture contents and lower hardness compared to bovine cheeses in agreement with our previous findings^22^. This difference was suggested to result from low κ-casein content in camel milk compared to bovine milk^5,47^. However, we have suggested that the high percentage of β-casein might also contribute to the soft and smooth nature of camel cheeses^41^. Moreover, the SDS-PAGE electropherograms (see below) suggest that some endogenous enzymes in camel milk might contribute to the softness of camel cheeses.

### Effects of camel chymosin and *Withania coagulans* on the physicochemical properties of camel and bovine cheeses

Table 5 presents the results of pH/acidity, fat, protein, and total solids in camel and bovine cheeses and whey. The camel milk cheeses were generally more acidic than bovine milk cheese. *Withania*-treated cheeses possessed higher acidity and lower pH in both camel and bovine cheeses and whey products. The high pH in the chymosin-treated cheeses may be associated with rapid coagulation of milk after the addition of chymosin, which triggers rapid casein cleaving and quick rearrangement of the caseins^48^. This results in larger pore spaces in the casein microstructure^22^ that enhance water drainage leading to increased pH due to lower activities of lactic acid bacteria in the dry gels^49^. In this study, we added calcium chloride (3%) to both kinds of milk as usually done during the preparation of bovine cheeses but it was reported that there is no observed improvement by adding calcium chloride with camel chymosin^50^. Table 5 shows significant differences between the different treatments with respect to the pH and acidity of the different cheeses. The importance of electrolyte balance for enzyme activities and casein coagulation during cheese-making is not well understood and deserves further investigations. The equilibria involving minerals (mainly calcium and magnesium but also sodium) and anions (such as phosphate, citrate, and acetate) are important determinants of casein micelle stability, pH, and enzyme activity^30^. Moreover, the high acidity and low pH of the camel compared with the bovine cheeses may be explained by an increased degree of proteolysis in camel milk cheeses, because proteolytic activities may produce peptides with acidifying effect (see below).

**Table 5:** Chemical compositions of camel and bovine milk cheeses and whey proteins (n = 3).

Treatment*	pH	Acidity (%)	Total solids (%)	Fat (%)	Protein (%)	pH	Acidity (%)	Total solids (%)	Fat (%)	Protein (%)
	Camel milk cheese					Camel milk whey				
PW	4.3 ± 0.03^d^	2.6 ± 0.04^a^	37.2 ± 0.16^f^	19.7 ± 0.06^e^	12.8 ± 0.06^de^	4.0 ± 0.01^e^	5.2 ± 0.04^a^	7.9 ± 0.06^a^	2.0 ± 0.01^a^	1.6 ± 0.04^c^
PC	4.9 ± 0.02^a^	0.8 ± 0.03^f^	45.5 ± 0.18^c^	28.4 ± 0.25^b^	12.6 ± 0.26^e^	4.5 ± 0.01^a^	3.6 ± 0.03^ g^	6.9 ± 0.051^d^	1.2 ± 0.00^c^	1.7 ± 0.02^bc^
LWLC	4.6 ± 0.02^b^	1.7 ± 0.025^d^	53.5 ± 0.16^a^	32.2 ± 0.13^a^	17.2 ± 0.23^a^	4.3 ± 0.02^c^	4.3 ± 0.04^d^	7.5 ± 0.087^b^	1.3 ± 0.01^d^	1.5 ± 0.02^d^
LWHC	4.7 ± 0.04^b^	1.7 ± 0.02^de^	49.5 ± 0.38^b^	28.6 ± 0.42^b^	15.6 ± 0.28^b^	4.4 ± 0.01^bc^	4.1 ± 0.011^e^	7.6 ± 0.07^b^	1.4 ± 0.00^d^	1.5 ± 0.02^d^
HWLC	4.4 ± 0.04^c^	2.4 ± 0.02^b^	45.8 ± 0.16^c^	23.5 ± 0.34^d^	14.6 ± 0.28^c^	4.2 ± 0.02^d^	4.6 ± 0.04^b^	7.7 ± 0.09^b^	1.4 ± 0.007^e^	1.5 ± 0.03^d^
MWMC	4.5 ± 0.03^c^	2.0 ± 0.04^c^	42.4 ± 0.39^d^	24.4 ± 0.26^c^	13.4 ± 0.24^d^	4.3 ± 0.01^d^	4.4 ± 0.04^c^	7.0 ± 0.10^c^	1.6 ± 0.01^c^	1.8 ± 0.05^a^
HWHC	4.7 ± 0.03^b^	1.6 ± 0.07^e^	40.5 ± 0.47^e^	17.9 ± 0.09^f^	12.7 ± 0.09^e^	4.4 ± 0.04^ab^	3.8 ± 0.04^f^	7.5 ± 0.10^b^	1.8 ± 0.02^b^	1.8 ± 0.01^ab^

Treatment*	pH	Acidity (%)	Total solids (%)	Fat (%)	Protein (%)	pH	Acidity (%)	Total solids (%)	Fat (%)	Protein (%)
	Bovine milk cheese					Bovine milk whey				
PW	4.6 ± 0.03^d^	1.2 ± 0.03^a^	51.6 ± 0.29^e^	29.4 ± 0.025^f^	18.3 ± 0.11^e^	4.3 ± 0.02^c^	3.5 ± 0.01^a^	6.9 ± 0.08^a^	1.2 ± 0.01^c^	1.3 ± 0.01^abc^
PC	5.3 ± 0.05^a^	0.2 ± 0.04^e^	68.9 ± 0.33^c^	41.4 ± 0.08^c^	19.5 ± 0.31^d^	4.6 ± 0.02^a^	2.0 ± 0.05^ g^	6.3 ± 0.03^b^	1.4 ± 0.01^a^	1.4 ± 0.01^a^
LWLC	4.9 ± 0.03^b^	0.5 ± 0.03^d^	77.2 ± 0.23^a^	40.1 ± 0.18^d^	22.3 ± 0.20^a^	4.4 ± 0.03^b^	2.5 ± 0.03^e^	6.0 ± 0.17^b^	1.3 ± 0.02^b^	1.3 ± 0.03^bc^
LWHC	4.8 ± 0.02^bc^	0.6 ± 0.04^d^	76.7 ± 0.22^a^	44.3 ± 0.08^a^	21.4 ± 0.23^b^	4.4 ± 0.02^c^	2.9 ± 0.02^d^	6.1 ± 0.16^b^	1.3 ± 0.02^b^	1.3 ± 0.02^c^
HWLC	4.8 ± 0.02^c^	1.0 ± 0.05^b^	74.8 ± 0.13^b^	42.7 ± 0.12^b^	21.0 ± 0.13^b^	4.3 ± 0.02^c^	3.3 ± 0.03^b^	6.1 ± 0.13^b^	1.3 ± 0.03^b^	1.3 ± 0.02^bc^
MWMC	4.8 ± 0.03^c^	0.9 ± 0.035^c^	68.2 ± 0.25^c^	41.4 ± 0.22^c^	20.1 ± 0.11^c^	4.4 ± 0.01^c^	3.1 ± 0.03^c^	6.3 ± 0.02^b^	1.3 ± 0.02^b^	1.3 ± 0.02^ab^
HWHC	5.2 ± 0.03^a^	0.3 ± 0.042^e^	59.2 ± 0.26^d^	33.2 ± 0.09^e^	20.5 ± 0.11^c^	4.6 ± 0.03^a^	2.2 ± 0.03^f^	6.8 ± 0.11^a^	1.2 ± 0.00^c^	1.3 ± 0.02^abc^

*Comparison was made between the different treatments for each cheese. Values within each column and each of the four categories (camel cheese, bovine cheese, camel whey, or bovine whey) carrying different superscript are statistically different (p < 0.05, n = 3 per treatment).

**Abbreviations are shown in Table 2.

**Figure 6:**
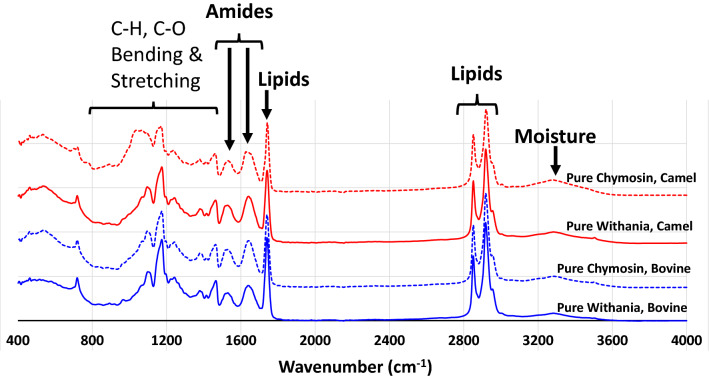
Fourier-transform infrared spectra of camel and bovine milk cheeses treated with pure *Withania* extract or chymosin (Experiment 2).

Cheeses made from a mixture of chymosin and *W. coagulans* had the highest total solids, protein, and fat. Furthermore, the results also show fat, protein, and total solid contents were significantly higher in the bovine milk cheeses than in the camel milk cheeses (Fig. 4), which is in agreement with previous findings^21,51^. The lower protein, fat, and total solid contents of camel cheeses and the higher total solid in whey shown in Table 5 may be associated with the softer nature of these cheeses. Another factor could be due to a lower concentration of κ-casein in camel milk compared to bovine milk (3.3% vs. 13%). The proportions of αs1-:αs2-:β-:κ-caseins in camel milk were 2.6:0.4:6.7:0.3, compared with 4:1:4:1 in bovine milk^52^. κ-Casein is known to enhance the coagulation properties of milk, leading to a denser casein matrix, which reduces the loss of fat and protein to the whey^53–55^. The κ-casein concentration and its relative proportion to αS1- and β-casein concentrations are usually low in poorly and non-coagulating bovine milk^56^. Determination of the exact contribution of the different caseins in camel milk to the texture of camel milk cheese remains a challenge. Figure 6 presents the spectra of cheeses at various wavelengths, ranging from 400 to 4000 cm^−1^. The spectra showed in both camel and bovine cheeses were similar to what was reported by previous researchers^57–59^.

### SDS-PAGE results on the proteolysis of camel and bovine milk cheeses

**Figure 7:**
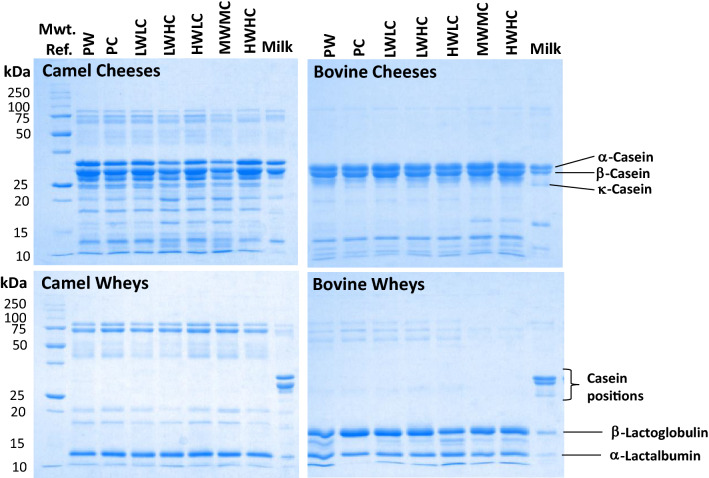
Sodium dodecyl sulfate–polyacrylamide gel electrophoresis of camel and bovine cheeses, wheys, and milk (Experiment 2).

The SDS-PAGE electropherograms showing differences in the protein and peptide profiles of camel and bovine cheeses and whey are presented in Fig. 7. It can be observed that camel cheeses show several low molecular weight bands compared to bovine cheeses suggesting that excessive proteolysis of caseins has occurred presumably catalyzed by endogenous enzymes such as plasmin in camel milk^60,61^. The proteolysis of β-CN by the natural milk proteases (plasmin) was successfully found in milk samples analysis before^62^. Thus, the high proportion of β-casein and possibly more active proteolytic activity in camel milk may lead to an increased level of proteolytic products. It was reported that high levels of β-casein affect milk coagulation causing softness of cheeses^63^. We have observed similar behavior in camel milk fermented by the lactic acid bacteria used to make yogurt (results not shown). Some of the low molecular weight peptides from camel milk cheese seem to migrate into the whey fraction explaining the low total solid content in camel milk cheeses and casein bands seen in the SDS-PAGE whey results.

## Conclusions

This study investigated the effect of different combinations of *W. coagulans* extract and camel chymosin on the yield, hardness, and total solids (protein, fat, and other solids) contents of cheeses prepared from camel and bovine kinds of milk. The results revealed that too high concentrations of the enzymes resulted in the production of soft cheeses. *W. coagulans* extract protease alone is not sufficient to produce good quality cheese especially camel milk cheese but a mixture of *W. coagulans* and camel chymosin produced better quality camel and bovine milk cheeses than chymosin alone. SDS-PAGE showed camel cheeses to more hydrolysis products compared to bovine cheeses suggesting possible participation of endogenous enzymes in camel milk. Further studies are needed to identify the enzyme(s) responsible for proteolytic activity in camel milk and their contribution to milk coagulation and cheese softness.
